# Is S-shaped kidney always a fusion anomaly? Radiological diagnosis of a new anatomical variant of a single kidney

**DOI:** 10.4103/0970-1591.57915

**Published:** 2009

**Authors:** Mayank Mohan Agarwal, Shrawan K. Singh, Arup K. Mandal

**Affiliations:** Department of Urology, Postgraduate Institute of Medical Education and Research, Chandigarh, India

**Keywords:** Calices, computed, hydronephrosis, kidney, pyelography, retrograde, tomography

## Abstract

The term ‘S-shaped kidney’ typically refers to a variant of crossed-fused ectopia in which the crossed kidney is fused with the lower pole of the orthotopic kidney maintaining its orientation resulting in medially facing upper-pelvis and laterally facing lower-one; no kidney is present in the other renal fossa.[1] We present rational diagnosis and management of a rare anatomic variant of a single kidney with S shaped anomaly.

## CASE REPORT

**Figure 1:**
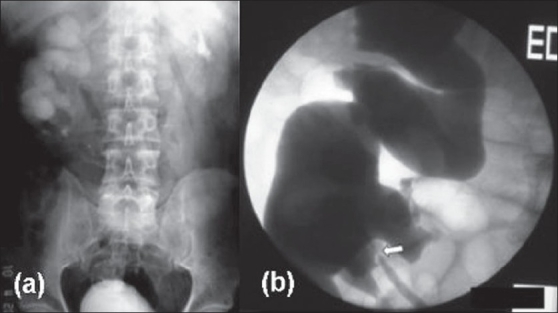
(a) Excretory-urography (EU) showing hydronephrosis with lateral upper calyces and medial lower calyces. Note the normally excreting anteriorly rotated left kidney; (b) Retrograde ureteropyelography showing laterally deviated ureter draining the whole of the pelvicalyceal system, with short-segment ureteropelvic junction obstruction

**Figure 2:**
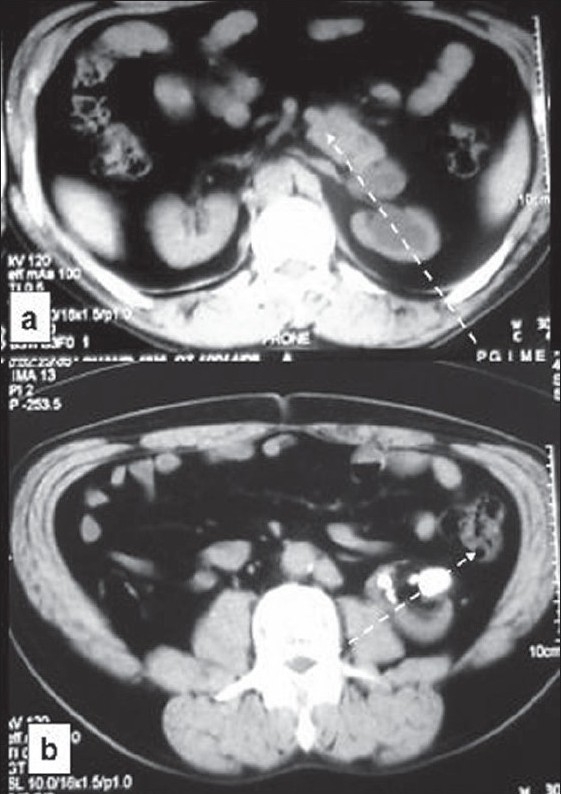
Computerized tomography, axial section through the (a) upper part of the right kidney showing anteromedial orientation of renal hilum and (b) the lower part anterolateral (axis depicted by broken arrow)

A 45 year old man presented with intermittent and colicky right flank pain which on Excretory-Urography (EU) was attributable to hydronephrotic right kidney with stones. Upper calyces were facing laterally and lower medially and ureteropelvic (UPJ) anatomy was obscure [Figure 1a]. Tc^99m-^ Diethylene triamine pentaacetic acid (DTPA) scan revealed a decreased perfusion and slow drainage in the right kidney with differential function of 45%. Computed tomography (CT) showed presence of a hydronephrotic S-shaped shaped right kidney with upper half of hilum facing anteromedially and lower half anterolaterally [Figure 2]. Retrograde ureterogram (RGU) immediately before surgical intervention revealed normal caliber single right ureter deviating laterally in the upper part opening through a narrow ureteropelvic junction (UPJ) into a laterally facing pelvis with medially facing lower calyces; the contrast also delineated the upper calyces, facing laterally [Figure 1b]. He underwent pyelolithotomy and Anderson-Hynes dismembered pyeloplasty. Postoperative course was uneventful; the patient remains asymptomatic and EU and DTPA at 3 and 6 months revealed improved drainage.

## DISCUSSION

The fused kidney (including s-shaped variant) has two independent collecting systems including the ureters. Generally, contralateral renal fossa is empty except in exceedingly rare circumstances of supernumerary kidney In the present case, the EU revealed calyces oriented in 2 directions which suggested presence of S-shaped kidney; however, presence of single Ureter draining the whole pelvicalyceal system as well as presence of normal contralateral kidney (which showed anterior malrotation) clinched the diagnosis of single S-shaped kidney rather than a fusion anomaly.

Even after extensive literature search, we did not come across any case of S-shaped shaped single kidney and therefore, it may represent the first such reported case. We postulate that it may be a rotational anomaly as evidenced by presence of arrested rotation in the contralateral kidney. It has been postulated that rotation actually is the result of unequal branching of successive orders of budding ureteral tree and since ventral buds are more, they induce differentiation of parenchyma more in ventral location giving an impression of medial rotation.[1] Malrotation may occur in case of insertion of ureteral bud in an atypical location of the renal blastema.[2] In the index case, an atypical lateral insertion may have induced disorderly branching and corresponding disordered parenchyma, leading to normal rotation of the upper half of the kidney and non-rotation / reverse-rotation of the lower half giving rise to the unique morphology.

## CONCLUSION

We describe the first case of an S-shaped single kidney which is different from the previously described S-shaped fusion anomaly.
